# Measuring functioning and disability in Korea: comparing general and dedicated surveys using the ICF as a reference framework

**DOI:** 10.1371/journal.pone.0323616

**Published:** 2025-05-12

**Authors:** Jiin Kim, Melissa Selb, Youngtae Cho, Wanho Kim, Carla Sabariego

**Affiliations:** 1 Faculty of Health Sciences and Medicine, University of Lucerne, Luzern, Switzerland; 2 National Rehabilitation Center, Ministry of Health and Welfare, Seoul, Republic of Korea; 3 ICF Research Branch, Nottwil, Switzerland; 4 Population Policy Research Center, Graduate School of Public Health, Seoul National University, Seoul, Republic of Korea; 5 Haeundae Sharing and Happiness Hospital, Busan, Republic of Korea; 6 Swiss Paraplegic Research, Nottwil, Switzerland; IRCCS Medea: Istituto di Ricovero e Cura a Carattere Scientifico Eugenio Medea, ITALY

## Abstract

**Introduction:**

Functioning is the third health indicator besides mortality and morbidity. Although Korea periodically collects functioning information, the functioning indicator has not been generated yet. This study aimed to evaluate how functioning information is currently collected in Korea’s health and disability surveys, especially in terms of comparability and comprehensiveness, using the International Classification of Functioning, Disability and Health (ICF) as a reference framework.

**Methods:**

Data sources included three health and disability surveys in Korea, namely the Population and Housing Census, the Korean National Health and Nutrition Examination Survey, and the Survey on the Living condition of Persons with Disabilities, and two international ICF-based surveys, the International Spinal Cord Injury Survey and the Model Disability Survey. Functioning information was linked by category to the ICF Generic-30 Set utilizing the ICF linking rules.

**Results:**

Three ICF categories — d450 walking; d510 washing oneself; d540 dressing – were covered by all the data sources. Excluding the Population and Housing Census, which inherently differs from the other data sources, all the surveys addressed six ICF categories — b152 emotional functions; b280 sensation of pain; d240 handling stress and other psychological demands; d450 walking; d510 washing oneself; d540 dressing. The ICF categories b152 emotional functions and b130 energy and drive functions were the most frequently covered across all the surveys. Despite of the common ICF categories, important differences in the operationalization of questions and response options were found.

**Conclusions:**

The overlap of content of health and disability surveys in Korea enables interoperability with other data sources. Available functioning data can be used for estimating the functioning indicator and metric, as recommended by WHO, which can serve as a valuable epidemiological indicator for complementing mortality and morbidity indicators and for estimating and monitoring rehabilitation or long-term care needs of the Korean population.

## Introduction

Functioning is increasingly considered a third health indicator besides mortality and morbidity [1]. Functioning, as defined by the World Health Organization (WHO) in the International Classification of Functioning, Disability and Health (ICF), is an overarching term that encompasses all body functions and structures, the activities an individual does on an everyday basis as well as participation in life situations and society [2,3], and takes into account both an individual’s intrinsic health condition and the surrounding [2] environmental context [4,5]. As an individual’s functioning declines, the person may experience disability, understood not only as a result of disease, disorder, or injury but as the outcome of the interaction between the health condition and the context of the person [3]. Moreover, disability, as defined in the ICF, spans a continuum from low to high levels [6–8]. Functioning enables us to comprehensively understand the biological and lived health [4] of the general population, including individuals traditionally considered as persons with disability and those without a certified disability but who experience problems in functioning due to health conditions, such as noncommunicable diseases (NCDs) and ageing [9].

Functioning information is essential for rehabilitation planning, monitoring, and evaluation [5]. WHO defines rehabilitation as “*a set of interventions designed to optimize functioning and reduce disability in individuals with health conditions in interaction with their environment*” [10]. It emphasizes that rehabilitation should be accessible to the entire population in need and across the lifespan [10], rather than being limited to people traditionally considered as persons with disability [9]. Based on data from the Global Burden of Disease (GBD) of 2019, it is estimated that around 2.4 billion individuals worldwide could potentially benefit from rehabilitation services due to their existing health conditions, most prominently due to sensory impairments (seeing and hearing loss) and musculoskeletal disorders [11]. According to the GBD data, an increase in rehabilitation need is related to the global demographic shift towards an ageing population [12]. Along with estimating the need for rehabilitation, reliable and comprehensive information on functioning is essential to inform the planning and delivery of services and to enable the monitoring and evaluation of rehabilitation programs [12,13].

According to WHO Rehabilitation 2030 Call for Action, functioning data can be collected from institutional sources (e.g., clinical records) as well as population level sources (e.g., censuses, surveys) to enhance the national health information system (HIS) with data that can inform rehabilitation planning [14,15]. In rehabilitation medicine in Korea, several validated tools for functioning assessment are used in the clinical setting [16–18]. These institutional-based data sources provide an excellent data base [15] about the populations who receive treatment in rehabilitation hospitals and clinics in Korea. However, given population ageing and the increase in NCDs, this data is ideally complemented with comprehensive population-level information on the functioning of the general and specific populations, including the ageing population and persons with non-communicable diseases. Population-based functioning information can also help enhance the quality of rehabilitation services at the national level and provide the basis for health and rehabilitation policy-making [14]. Functioning information obtained from both data sources can be used to capture all dimensions of the impact of health conditions (injury, disease, ageing) on an individual’s lived experience [14].

Korea has rich health data at the national level. Nevertheless, the functioning data collected is neither fully comparable across regions of the country nor comparable with functioning data collected internationally. For example, the Korean National Disability Survey, also called Survey on the Living condition of Persons with Disabilities (SLPD) [19], was not developed based on an international standard like the ICF [20], and unlike WHO’s Model Disability Survey (MDS) [21], it is not designed to collect functioning information of the general population [22]. The MDS, a functioning and disability survey tool based on the ICF, provides comprehensive information about the levels of disability in a population and their determinants, while the SLPD focuses mainly on disabling diseases and selected diagnoses, and targets only people who are registered in the national disability registration system [23]. This means that the population experiencing mild to moderate functioning limitations or those with a health condition but not considered disabled, are not covered by the survey. This limits the usefulness of the survey for planning rehabilitation programs for all those in need.

The Korean government has come to recognize the importance of collecting functioning information according to international standards. In 2016, Statistics Korea established the Korean Standard Classification of Functioning, Disability and Health (KCF), which is rooted in the ICF but translated into Korean [24]. Two years later, Statistics Korea conducted a pilot study of 300 households residing in Seoul using the MDS [25]. After the pilot study, questions such as ‘limitations on activities’ and ‘care for persons with activity limitations’ were incorporated in the Population and Housing Census (hereafter referred to as the Census) in 2020 [26,27]. Unfortunately, the questions contained in the Census are not comprehensive enough to enable an ample estimate an overall functioning indicator, and adding questions is not possible due to restrictions on the length of the census. Another example of Korea’s efforts to incorporate functioning information in disability data collection, the National Rehabilitation Center, the government agency that provides rehabilitation services and is responsible for rehabilitation research and training in Korea, has been participating in the International Spinal Cord Injury (InSCI) survey since 2017 with the aim of collecting internationally comparable functioning information on spinal cord injury (SCI) [28,29]. Lastly, the Korean National Health and Nutrition Examination Survey (KNHANES) [30], which is designed to investigate people’s health behavior, the prevalence of chronic disease, and health risk factors, includes as well functioning-related questions [31].

Given the importance of collecting functioning information for rehabilitation program planning at the national level and the advantages of estimating a functioning indicator that can complement data about mortality and morbidity, it is essential to examine to what extent functioning information currently collected in Korea with different data sources is comparable and interoperable. Structured mappings of the content of national and international surveys and censuses using the ICF as a reference framework are important for unveiling their content and comparability. The objective of this paper is to evaluate how functioning information is currently collected in Korea’s health and disability surveys, especially in terms of their comparability across data sources, and comprehensiveness, i.e., to what extent ICF categories of the ICF Generic-30 Set*, which encompasses the data needed for rehabilitation care and planning are covered. This work supports a better understanding of each instrument and can inform efforts towards a minimum harmonization of functioning data collection as a first step towards interoperability: the ability to connect, exchange, and utilized information across different institutions and systems [32].

## Methods

### Data resources

Five data sources implemented in Korea, the Korean Census, KNHANES, SLPD, InSCI questionnaire, and MDS, were used in this study and described in detail below.

The Census is conducted every five years to identify population and household characteristics to assist in establishing and evaluating various economic and social development plans and research [26]. Korea conducts both a complete enumeration census using administrative data, known as registration census, and a field survey involving sample households (20%) through on-site visits [26]. In the Census conducted in 2015, data about the presence or absence of physical and mental constraints that have continued for or are expected to continue for 6 months or more (disability) was collected [26]. However, in the 2020 Census, the questions were modified in accordance with the UN Sustainable Development Goals (SDGs) recommendation to investigate whether there was a disability at a specific point in time regardless of the how long the disability was present, and the duration of disability (6 months) was investigated as an additional question [26]. Additionally, the two questions surveyed, ‘physical and mental constraints’ and ‘constraints in daily life and social activities’, were integrated into one question called ‘limitations on activities’ in the 2020 census [26].

The KNHANES is a nationwide health and nutrition survey conducted every year to generate representative and reliable statistics on the health status, health behavior, and the food and nutrition intake of the Korean population [31]. The survey results are used to guide health policies, such as goal-setting and evaluation of national health plans as well as development of health promotion programs. The KNHANES consists of three parts. The first part encompasses a household survey, health interview survey, and health behavior survey. The household survey asks one adult ≥19 years old per household about the general characteristics of the household. The health interview survey includes questions about morbidity, use of medical services, activity restrictions, education and economic status, and physical activity. The health behavior survey contains questions about smoking, drinking behavior, mental health, safety awareness, and oral health. The second part is a physical examination which comprises of basic tests (diagnosis of disease such as stroke, diabetes, and medication use), blood pressure and physical measurements (e.g., height, weight), and blood and urine tests [31]. The nutrition survey is the last part; it asks about dietary habits and the consumption of dietary supplements. Our study used only the health and health behavior survey of the KNHANES 2020. Seven Activities of Daily Living (ADL) questions and ten Instrumental Activities of Daily Living (IADL) questions were included in the 1998 and 2005 waves, but these items were excluded in the 2020 survey [31].

The SLPD aims to identify the population of persons with disabilities and the prevalence of disabilities in Korea based on the Act on Welfare of Persons with Disabilities [23]. The SLPD is conducted every three years. We used the 2020 version in the present study, which was an exceptional year due to the COVID-19 pandemic and utilized a national registry of people with disabilities to select the sample [23]. In the previous SLPD conducted until 2017, the first step involved conducting a preliminary survey on general households using the Census districts, and if individuals with disabilities were identified in those households, a disability survey was carried out [23]. The survey investigates the living conditions and welfare needs of persons with disabilities to inform the establishment and implementation of short- and long-term welfare policies. The survey is divided into three parts. The first part asks questions about socio-demographics and general characteristics of the disability, such as year of disability registration and type of registered disability. The second part asks more detailed questions about the disability, such as type, onset and cause of disability. The last part includes questions about healthcare, support needs in daily life, assistive devices, social activities, welfare services, and financial status, etc.

The InSCI survey was developed as the first step of the Learning Health System for Spinal Cord Injury (LHS-SCI) to generate comprehensive data on SCI, and to understand the lived experience of people with SCI living worldwide [28]. The InSCI questionnaire comprises questions that cover 47 ICF categories included in the ICF Generic-30 Set, and the Brief ICF core set for SCI for the long-term context [28,33,34]. The questionnaire consists of 11 components, including personal information, lesion characteristics, energy and feelings, health problems, activity and participation, independence in activity of daily living, work, environmental factors, health care services, personal factors, and quality of life and general health [35]. The first round of the InSCI survey was conducted in 22 countries between 2017 and 2019 [36], and the second round is currently being conducted (2023–2024) with an updated questionnaire. In the present study, the 2017 version of the InSCI questionnaire containing 125 items was employed [37].

The MDS was developed based on the ICF to collect comprehensive and comparable functioning and disability data [22] and to monitor the UN Convention on the Rights of Persons with Disabilities (CRPD) [38]. The MDS collects data on all dimensions of functioning and disability, including impairments, activity limitations, participation restrictions, and environmental factors that affect an individual’s full participation. The full version of MDS with 337 questions was used in the 2018 pilot study in Korea [21,25], and the same questionnaire was used in the present study.

### Descriptive comparison and linking to the ICF

First, the tools were compared descriptively. The MDS and InSCI surveys are in English. To make the Korean surveys comparable with the InSCI and MDS, we translated the KNHANES and SLPD into English. TA researcher who had previously participated in translating the Korean version of the MDS [25] and developing the simple description of the Korean version of the ICF Generic-30 Set [39] translated the questionnaire. Afterwards, two researchers (JK, MS) proficient in the ICF, one of whom is a native English speaker (MS), reviewed the translation. The translation process also included considering the purpose of the survey as well as the response options. For instance, in the ‘Activity limitation and Quality of life’ chapter of the KNHANES, question “4-1. exercise ability” could have been translated as either “exercise ability” or “motion ability” when directly translating from Korean to English. However, considering the response options and the intent of the question, “exercise ability” was deemed the most appropriate translation. The English version of the Census was provided by Statistics Korea. In a first step, we described the purpose, use, and target population of the survey, survey cycle, and the composition of each survey.

To find out which functioning categories are covered by each data source, we linked the questions of the surveys to the corresponding ICF category using the ICF linking rules. The ICF linking rules was developed to enhance the comparability of health information, ensuring that information is available in a consistent manner [40]. When aggregating and integrating health information from different sources, comparability is challenged by the heterogeneity in both content and mode of data collection [40]. Thus, the ICF linking rules entails the identification of the perspectives that each question reflects (i.e., descriptive-general, -capacity, -performance; appraisal; need and dependency), the categorization of the corresponding response options (i.e., intensity; frequency; duration; confirmation or agreement), identification of main concepts and lastly, the identification of the precise ICF category that represents the each main concept [40]. Even if two surveys collect information on the same category, each question may collect this information from different perspectives [40]. Therefore, for a clear data comparison, it is essential to clarify both the category and the perspective of collecting information on a given category. Furthermore, the decision about the precise ICF category is informed by the categorization of the response options as well as by the context of the information [40], such as sections of a survey containing questions with an overarching construct (represented by the section heading).

In this study, we employed the ICF linking rules in a slightly modified fashion. That is, instead of linking each survey question freely to any ICF, we identified and documented word for word the questions from the respective survey that were deemed, if any, as corresponding to the categories of the ICF Generic-30 Set. The ICF Generic-30 Set is a minimal set for assessing functioning and disability in clinical settings and population-based health surveys, consisting of 30 categories from the ICF components of body functions and activities and participation [33,41]. We used the ICF Generic-30 Set as a reference framework for the following reasons: 1) it is practical because it is compressed into essential categories compared to the entire ICF classification, 2) it includes the minimal generic set of functioning and health (ICF Generic-7 Set), consisting of 7 categories, designed to capture the experience of individuals and populations regarding functioning and health [42], 3) it is one of the few non-disease specific ICF core sets suitable for cross-conditions and cross-surveys comparisons and 4) there is a Korean language/country-specific version of the simple description of the ICF Generic-30 Set, which can increase the accessibility of our results for policy makers and stakeholders who are not familiar with the ICF [39]. As previously mentioned, the Generic Set also served as the basis for the InSCI questionnaire [28] and was considered in the development of the MDS [33,43]. It can also be employed in monitoring the impact of rehabilitation interventions at all levels of healthcare [41].

**Fig 1:**
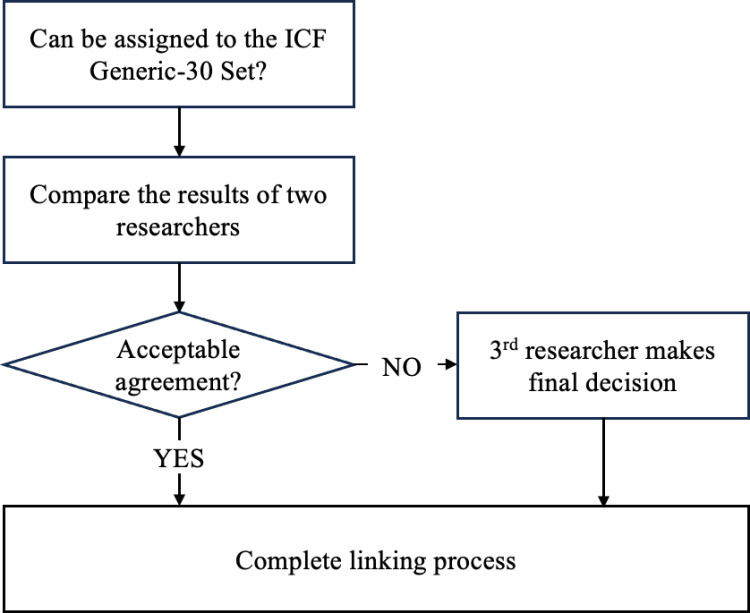
Linking process tree.

The other elements of the standard linking procedure, i.e., that two researchers [40] first performed the linking procedure independently, including the documentation of the perspective and categorization of the response options, before discussing their results to arrive at a consensus on the final linking. Fig 1 shows the modified linking process. The linking procedure was performed according to the following criteria:

- questions targeting difficulties, limitations and problems in functioning were included.
- if the question includes two or more concepts, the main concept and additional concepts are linked to each ICF category. If the perspective of the question is descriptive, the researcher documented whether the question is about capacity or performance. If this is not clear, then the researchers only documented the question as “descriptive-general”.

If there are any particularities that occurred during the linking process, researchers recorded in the research diary to enhance the transparency and consistency of the process [40]. Only the three Korean surveys were linked to the ICF Generic-30 Set as the InSCI and MDS are ICF-based surveys.

## Results

General characteristics of the five data sources, such as target population and survey cycle, along with information on health conditions and health services collected in each survey, are shown in Table 1. Information on functioning was collected in three modules (Activity limitation and quality of life; Sleep health; Mental health) of the KNHANES, four modules (Disability characteristic; Healthcare and health; Daily activity support; Social, culture, and leisure activity) of the SLPD and the module on activity limitations of the Census.

**Table 1:** Overview of the five data sources (Census, KNHANES, SLPD, MDS, InSCI).

	Census	KNHANES	SLPD	MDS	InSCI
Development and promotion	Statistics Korea	Korea Disease Control and Prevention Agency (KDCA)	Korean Ministry of Health and Welfare, Korea Institute for Health and Social Affairs (KIHASA)	WHO, World Bank	WHO, International Spinal Cord Society (ISCOS), International Society of Physical and Rehabilitation Medicine (ISPRM)
Country	South Korea	South Korea	South Korea	International	International
Scope	National survey on population and household	National survey on health risk factors, prevalence of major chronic disease and health behavior	National survey on people with disabilities	National/regional survey on disability and health	National/community survey on the lived experience of people with spinal cord injury
Target population	Population of all ages	Population only adults	Population of all ages who are registered in the national disability registration system	Population of all ages	Adults with spinal cord injury and experience living in the community
Survey cycle	every 5 years	every year	every 3 years	every 5 years	every 5 years
Collection mode	Interview	Interview	Interview	Interview	Interview
Assessment	Self-reported	Self-reported	Self-reported/proxy	Self-reported/proxy	Self-reported
Modules for functioning assessment	1 (activity limitation)	3 (Activity limitation and quality of life; Sleep health; Mental health)	4 (Disability characteristic; Healthcare and health; Daily activity support; Social, culture, leisure activity)	3 (Functioning; Health condition; Well-being)	5 (Energy and feeling; Health problems; Activity and participation; Independence in activities of daily living; Work)
**HEALTH CONDITIONS**					
Content	Not applicable	hypertension; dyslipidemia; stroke; myocardial infarction; angina; osteoarthritis; rheumatoid arthritis; osteoporosis; tuberculosis; asthma; thyroid disease; diabetes; cancer; depression; atopic dermatitis; allergic rhinitis; paranasal sinusitis; otitis media; cataract; glaucoma; macular degeneration; diabetic retinopathy; kidney disease; hepatitis; cirrhosis; gout; oral health	physical disability; brain injury; vison; hearing; language; intellectual; autism; mental; renal; cardiac; respiratory; hepatopathy; facial; entero-/urostomy; epilepsy (discomfort part; causal of disability; main diagnosis of disability); chronic disease (e.g., high blood pressure, stroke, diabetes, myocardial infarction....)	vision; hearing; hypertension; diabetes; arthritis; heart disease; chronic bronchitis; asthma; pack pain; migraine; stroke; depress; anxiety; leprosy; amputation; polio; gastritis; cancer; trauma; dementia; kidney disease; skin disease; tuberculosis; mental disorder; sleep problem; tinnitus	sleep; bowel and bladder dysfunction; sexual dysfunction; contractures; muscle spasms; pressure sores; respiratory problem; injury caused by loss of sensation; circulatory problem; autonomic dysreflexia; postural hypotension; pain
Kind of information collected	Not applicable	Survey asks whether s/he has a diagnosed condition and currently acute, and whether the current treatment status.	Survey asks what type and level of the registered disability as well as about accompanying symptoms.	Survey asks if s/he has a health condition, and whether s/he has been given a diagnosis, medication and treatment for the health condition.	Survey asks how much of a problem s/he has caused by health problem, and whether s/he receives treatment for it.
Time frame	Not applicable	the year of first diagnosed; current status	the year of disability registration; current status	last 30 days; last 12 months	last 3 months
Question about general appraisal of health?	Not applicable	How do you feel about *household member name’s* health in general?	How do you feel about your health in general?	Including your physical and your mental health. In general, how would you rate your health today?	In general, would you say your health is…?; Compared to one year ago, how would you rate your health in general?
Response options	Not applicable	very good; good; normal; bad; very bad	very good; good; normal; bad; very bad	very good; good; moderate; bad; very bad	excellent; very good; good; fair; poor. Or much better; somewhat better; about the same; somewhat worse; much worse
**HEALTH SERVICES**					
Content	Not applicable	medical service use; vaccination and medical checkup; hospitalization; outpatient use; oral health	health insurance; regular treatment; medical checkup; being hospitalized; medical staff and facility; welfare services; rehabilitation treatment	inpatient care; outpatient care and care at home; responsiveness of health care system	healthcare provider; use of hospital and rehabilitation facility
Kind of information collected	Not applicable	Survey asks whether the respondent has needed medical service. If they answered ‘yes’ and didn’t get the service, s/he is then asked why s/he didn’t get medical service.	Survey asks type of health insurance, status of receiving treatment, type of medical checkup, and the reason if s/he didn’t receive medical checkup. Also, it asks experience of medical services (facility, outpatient, hospitalization) and welfare services.	Survey asks how many times s/he used health care service in the last 12 months. It asks the reason if s/he didn’t receive service and rate of the experience of health care system that s/he recently use.	Survey asks about the experience of using healthcare provider visiting service and medical facility service. The respondent rates their satisfaction. It also asks the reason if s/he didn’t receive medical service.
Time frame	Not applicable	last year; last 2 years	current status; last 2 years	last 3 years; last 12 months; for her/his last visit	last 12 months; for her/his last visit

Table 2 displays the frequency of questions from four perspectives and four response options when surveys are linked to the Generic-30 Set according to the ICF linking rules. This elucidates the focal aspects of each survey in collecting information on functioning. Most of the perspective of the included questions are general descriptive in nature or about need or dependency. Items related to capacity assessment were identified in all five surveys, while items addressing performance were only included in the MDS. The SLPD stood out with 18 items assessing need and dependency. In terms of the response options, the intensity or severity of a problem (i.e., how much of a problem a person has) was the most common, followed by confirmation (i.e., if a problem is present) and frequency (i.e., how frequently a person has a problem).

**Table 2:** Frequency of perspectives and response options per question, and cutoff for disability.

	Census	KNHANES	SLPD	MDS	InSCI
**PERSPECTIVE OF INCLUDED QUESTONS (# of questions)**					
Descriptive - General assessment	0	12	5	1	25
Descriptive - Capacity assessment	1	7	2	13	6
Descriptive - Performance assessment	0	0	0	27	0
Need and dependency assessment	3	2	18	0	15
Functioning questions time frames	only at the interview time	only at the interview time; past 2 weeks; past month	only at the interview time; past year	last 30 days	last 4 weeks; last 3 months; last week; only at the interview time
**CATEGORIZATION OF THE RESPONSE OPTIONS OF INCLUDED QUESTIONS (# of questions)**					
Intensity	0	8	21	41	33
Frequency	0	8	0	0	9
Duration	0	0	0	0	0
Confirmation or agreement	4	5	4	0	4
Scale	ordinal 1–2 (yes; no)	ordinal 1–2 (yes; no); 1–3 (no problem; cannot do or severe problem); 1–4 (not at all; almost every day); 1–4(feel very much; hardly feel)	ordinal 1–2 (yes; no); 1–5 (very discomfort; not discomfort at all); 1–4 (very much; hardly); 1–4 (no support needed; full support needed); 1–5 (can do by myself; almost everything requires support); 1–4 (very uncomfortable; not uncomfortable at all); 1–4 (very difficult; not difficult at all)	ordinal 1–5 (none; extreme or unable); 1–4 (no, no difficulty; cannot do at all); 1–5 (not at all; completely)	ordinal 1–5 (all of the time; none of the time); 1–5 (no problem; extreme problem); 0–10 (no pain; pain as bad as you can imagine); 1–5 (without any difficulty; unable to do); 1–5 (need total assistance; completely independent); 1–4 (need total assistance; independent without adaptive devices)
**SCORING OF FUNCTIONING AND DISABILITY**					
Cutoff for disability	not defined	not defined	not defined, but the questions are predefined according to the types of disability	no disability (score < Mean - 1SD or score = 0); mild disability (Mean - 1SD < Score < Mean); moderate disability (Mean < Score < Mean + 1SD); severe disability (Score ≥ Mean + 1SD)	not defined, but a score of 4 or higher can be regarded as having significant functional limitation

Table 3 shows how often (i.e., the number of items identified) categories of the ICF Generic-30 Set were addressed in each survey. Three categories of the Generic-30 Set (d450 walking; d510 washing oneself; d540 dressing) were included in all instruments. Excluding the Census, which is by default different from the other surveys, a total of six ICF categories — b152 emotional functions; b280 sensation of pain; d240 handling stress and other psychological demands; d450 walking; d510 washing oneself; d540 dressing — were common across all the surveys.

**Table 3:** Frequencies showing how often the ICF Generic-30 Set categories are addressed in the surveys.

ICF Code	ICF Title	Census	KNHANES	SLPD	MDS	InSCI
Total (absolute number)		4	21	25	41	46
b130	Energy and drive functions (G)	–	5	–	2	4
b134	Sleep functions	–	2	–	2	1
b152	Emotional functions (G)	–	2	1	4	5
b280	Sensation of pain (G)	–	1	2	2	2
b455	Exercise tolerance functions	–	3	–	2	1
b620	Urination functions	–	–	1	–	1
b640	Sexual functions	–	–	–	–	1
b710	Mobility of joint functions	–	–	1	–	1
b730	Muscle power functions	–	–	–	–	–
d230	Carrying out daily routine (G)	–	2	1	–	1
d240	Handling stress and other psychological demands	–	1	1	2	1
d410	Changing basic body position	–	–	1	1	3
d415	Maintaining a body position	–	–	1	1	4
d420	Transferring oneself	–	–	1	–	1
**d450**	**Walking (G)**	**1**	**1**	**1**	**3**	**1**
d455	Moving around (G)	–	–	1	3	1
d465	Moving around using equipment	–	–	–	–	1
d470	Using transportation	–	–	2	1	1
**d510**	**Washing oneself**	**1**	**1**	**1**	**2**	**2**
d520	Caring for body parts	–	–	1	1	1
d530	Toileting	–	–	2	2	3
**d540**	**Dressing**	**1**	**1**	**1**	**2**	**2**
d550	Eating	1	–	2	1	1
d570	Looking after one’s health	–	–	1	1	1
d640	Doing housework	–	–	2	2	1
d660	Assisting others	–	–	–	2	1
d710	Basic interpersonal interactions	–	–	–	–	1
d770	Intimate relationships	–	–	–	1	1
d850	Remunerative employment (G)	–	2	–	3	1
d920	Recreation and leisure	–	–	1	1	1

Legend: Bold-marked rows indicate a category that was mapped across all the surveys; (G) categories included in the ICF Generic-7 Set

As for the ICF category with the highest frequency across all the surveys, b152 emotional functions was identified most often (12 times), followed by b130 energy and drive functions (11 times). These two categories also belong to the ICF Generic-7 Set, the ICF set considered as the minimal standard for assessing and reporting functioning of any population and context, irrespective of the presence of a health condition [42]. The categories of the Generic-30 Set that not addressed in the Korean surveys were b640 sexual functions, b730 muscle power functions, d465 moving around using equipment, d660 Assisting others, d710 basic interpersonal interactions, and d770 intimate relationships.

While some ICF categories are common across the surveys, the way these categories are operationalized into questions and their corresponding response options varies between the surveys. This variance plays a significant role on to what extent the collected functioning information is comparable and interoperable across surveys. Table 4 shows the question and the corresponding response options from each survey that was found to reflect selected categories of the ICF Generic-30 Set. For example, when it comes to the item related to d450 walking, the MDS asks about “*how much of problem or difficulty the person has in walking*”, addressing the intensity of difficulty from both a capacity and performance perspective, while the KNHANES asks about the degree of difficulty and the Census focuses on confirming the presence of difficulty only. The InSCI and SLPD, which concentrate on SCI and persons with disabilities, ask how much help is needed to walk independently from a need/dependency perspective.

**Table 4:** Commonly identified ICF Generic-30 Set categories: survey questions and response options.

ICF Code	MDS question	InSCI question	Census question	Census response options	KNHANES question	KNHANES response options	SLPD question	SLPD response options
b152 Emotional functions	I4024. How much of a problem do you have with feeling sad, low or depressed?	16. How much of the time during the last 4 weeks...Have you been very nervous?			A4-5. Please check the one that best describe your health today. Anxiety/depression	1. I am not anxious or depressed; 2. I am somewhat anxious or depressed; 3. I am very anxious or depressed	H32. During the past year, have you felt sad or hopeless enough to interfere with your daily life for more than 2 weeks in a row?	1. yes; 2. no
	I4025. How much of a problem do you have with feeling worried, nervous, or anxious?	17. How much of the time during the last 4 weeks...Have you felt so down in the dumps that nothing could cheer you up?			M1-2. In the past 2 weeks, how often have you suffered from the symptoms listed below? feeling subdued, depressed, or hopeless	1. not at all; 2. for several days; 3. over a week; 4. almost everyday		
	I5013. To what extent do you feel sad, low or depressed because of your health?	18. How much of the time during the last 4 weeks...Have you felt calm and peaceful?						
	I5014. To what extent do you feel worried, nervous or anxious because of your health?	20. How much of the time during the last 4 weeks...Have you felt downhearted and depressed?						
		22. How much of the time during the last 4 weeks...Have you been happy?						
b280 Sensation of pain	I4019. How much of a problem is having pain in your day-to-day life for you?	37. For the following health problems please rate how much of a problem it was for you in the last 3 months. Pain			A4-4. Please check the one that best describe your health today. Pain/discomfort	1. I have no pain or discomfort; 2. I have some pain or discomfort; 3. I have very severe pain or discomfort	P2 & B2. You said you have discomfort in your arms, legs, or back. Where do you feel discomfort?	upper limbs (1. right; 2. left; 3. both), lower limbs (1. right; 2. left; 3. both), spine
	I5017. How much bodily aches or pains do you have?	38. Please rate your pain by circling the number that best describes your pain at its worst in the last week.					B3-4. Do you have the following accompanying symptoms and disabilities? Pain	1. yes; 2. no
d240 Handling stress and other psychological demands	I4030. How much of a problem is handling stress, such as controlling the important things in your life?	42. In the last 4 weeks, how much of a problem have you had handling stress?			M1. How much stress do you feel in your daily life?	1. feel very much; 2. feel a lot; 3. feel a little; 4. hardly feel	H31. How much stress do you feel in your daily life?	1. very much; 2. a lot; 3. little; 4. hardly
	I4031. How much of a problem is coping with all the things you have to do?							
d450 Walking	I4004. How much of a problem is walking a short distance such as a 100m for you?	70, Please check the box that reflects your current situation. Moving around moderate distances (10–100 meters). I walk moderate distances and I*...(how much do you need help)*	11. Do you have any of the following conditions due to health issues? Difficulty walking or climbing stairs?	1. Yes; 2. No	A4-1. Please check the one that best describe your health today. Exercise ability	1. I have no problem walking; 2. I have some problem walking; 3. I have to lie down all day	D1-9. To what extent are you able to perform the following routine movements on your own? Walking	1. no support needed; 2. need some support; 3. significant support needed; 4. full support needed
	I4005. How much of a problem is walking a kilometer for you?							
	WG3. Do you have difficulty walking or climbing steps?							
d510 Washing oneself	WG5. Do you have difficulty (with self-care such as) washing all over or dressing?	60. Please check the box that reflects your current situation. Washing your upper body and head *(how much do you need help)*	11. Do you have any of the following conditions due to health issues? Difficulty getting dressed/undressed, bathing, eating on your own?	1. Yes; 2. No	A4-2. Please check the one that best describe your health today. Self-care	1. I have no problem taking a bath or getting dressed; 2. I have some problems taking a bath or getting dressed myself; 3. I cannot take a bath or get dressed myself	D1-2. To what extent are you able to perform the following routine movements on your own? Take a bath	1. no support needed; 2. need some support; 3. significant support needed; 4. full support needed
	I4010. How much of a problem is being clean and dressed?	61. Please check the box that reflects your current situation. Washing your lower body *(how much do you need help)*						
d540 Dressing	I4010. How much of a problem is being clean and dressed?	62. Please check the box that reflects your current situation. Dressing your upper body *(how much do you need help)*	11. Do you have any of the following conditions due to health issues? Difficulty getting dressed/undressed, bathing, eating on your own?	1. Yes; 2. No	A4-2. Please check the one that best describe your health today. Self-care	1. I have no problem taking a bath or getting dressed; 2. I have some problems taking a bath or getting dressed myself; 3. I cannot take a bath or get dressed myself	D1-1. To what extent are you able to perform the following routine movements on your own? Change clothes	1. no support needed; 2. need some support; 3. significant support needed; 4. full support needed
	WG5. Do you have difficulty (with self-care such as) washing all over or dressing?	63. Please check the box that reflects your current situation. Dressing the lower body *(how much do you need help)*						

Legend: In the KNHANES, ‘A’ means Activity limitation and Quality of life module, and ‘M’ means Mental health module; In the SLPD, ‘H’ means Healthcare and health chapter. ‘P’ means Physical Disability chapter, ‘B’ means Brain injury disability chapter, and ‘D’ means Daily activity support chapter

## Discussion

Our study evaluated how functioning information is currently collected in Korea’s health and disability surveys, especially in terms of comparability and comprehensiveness, using the ICF as a reference framework. A total of 11 ICF Generic-30 Set categories were linked in the KNHANES, and 20 and 4 categories were identified in the SLPD and Census, respectively. In terms of comparability, the number of questions addressing common ICF categories across the data sources was three, including the Census, and six, excluding the Census, which generally includes very brief modules of a range of topics. In terms of comprehensiveness, ca. 80% of the content of the ICF Generic Set was covered by the Korean surveys.

Our study is comparable to a study by Lenildo de Moura et al. (2017) comparing population Brazilian data from different sources including the MDS and the Brazilian National Health Survey with the purpose of informing the development of a new ICF-based disability module for the latter [44] and to the study Gretchen Swanson et al. (2003), who back-coded specific survey questions from the national disability survey of 5 countries’—Canada, France, Netherlands, South Africa, and the USA— and developed a method to compare their data across countries [45].

To make most use of national functioning data, minimum standards in terms of which and how data is collected across different data sources are essential. The interoperability and comparability of functioning information may play a crucial role in informing decision-making regarding rehabilitation programs and policies [14], while also strengthening data quality of the health information system [46]. The ICF serves as a universal framework [46] and is a recognized widely as the most suitable one for documenting information on functioning [47]: its internationally accepted language and coding system enable seamless data comparison across countries and healthcare settings. Accordingly, the results of this study show that functioning information collected periodically in Korea ensures a minimum level of interoperability. This means that functioning indicators can be generated from available data. As recommended by the WHO, traditionally collected national data should be modified and expanded to meet the informational needs of rehabilitation planning [14].

Given the distinctive context of Korea, which is facing the world’s lowest fertility rate, while the life expectancy is predicted to be the world’s highest, a functioning indicator that complements information on mortality and morbidity is very much needed [47]. The study by Sophie L. W. Spoorenberg et al. (2015), which developed the Geriatric ICF Core Set, found that the most frequently reported ICF categories in older adults were d710 Mobility of joint functions, b152 Emotional functions, b455 Exercise tolerance functions, d450 Walking, and d410 Changing basic body positions [48]. Our result shows that d450 Walking is included in all three Korean surveys, b152 Emotional functions is included in the KNHANES and SLPD, and the rest are included in at least one survey. This means that functioning information currently being collected in the national health and disability survey can provide the foundational information needed to address the challenges associated with an ageing population. The definitions of functioning and disability proposed in the ICF are based on three assumptions: 1) disability is a universal human experience, 2) disability is etiologically neutral, and 3) disability is a continuous phenomenon [22]. Nevertheless, we found that the distinction based on disability types based on medical criteria was still used in the version of the SLPD survey that we mapped. Particularly noteworthy is the absence of the performance – what an individual does in his or her current environment [2] – assessment perspective questions in the Korean surveys, indicating a need for greater consideration of the impact of social and environmental factors on functioning limitations.

In addition to having a functioning indicator, developing a common metric would also enable the comparison of different populations of people in Korea, e.g., general population versus the disabled population. Having comparable functioning data across surveys opens the possibility of generating a common functioning metric, and this study contributes to generating such comparable functioning by evaluating the extent Korean health and disability survey collect functioning information. The following studies showcase how a common metric can be used. Cieza et al. (2015) harmonized information on functioning domains collected from two large populations surveys and used this information to build a common functioning metric for the purpose of quantifying and comparing functioning between two countries [49]. Similarly, Caballero et al. (2017) created a functioning score and metric that allowed the comparison of health ageing trajectories using data from more than 130,000 individuals that participated in a large range of cohort studies and waves of data collection [50]. Both studies pinpointed anchor functioning questions obtained from self-report, and systematically utilized Item Response Theory (IRT) or a combination of IRT and machine learning to construct a metric of functioning. Our study shows that generating a comparable functioning metric using common functioning domains as anchor questions is possible for Korea with the KNHANES and SLPD, which share the ICF categories b152 emotional functions, b280 sensation of pain, d240 handling stress and other psychological demands, d450 walking, d510 washing oneself, and 540 dressing. This selection might seem too small but Oberhauser C et al. demonstrated that it is possible to develop a valid and reliable health metric based on functioning for tracking and comparing population health using data from just six ICF domains of the minimal generic set—energy and drive functions, emotional functions, sensation of pain, carrying out daily routine, walking, and moving around [51]. As the present study recorded the inclusion of questions corresponding to this minimal set in both the KNHANES and InSCI, this information can be readily used for generating a Korean common health metric based on functioning which allows for the comparison of populations addressed in different surveys, for instance between the general Korean population and persons with disabilities or persons with SCI living in Korea. Our findings have established the feasibility of developing such a metric, which we anticipate will play a pivotal role in providing valuable data for estimating and monitoring rehabilitation or long-term care needs of the rapidly ageing population.

Our study has several limitations. The first is that we only used the ICF Generic-30 Set for linking, not the entire ICF classification, thereby excluding environmental factors and body structures categories. To compensate for this, Table 1 outlines the health conditions and health services questions included in each survey, but only in a limited way. The mapping revealed four ICF categories (b140 Attention functions, b144 Memory functions, d3 Communication, d451 Going up and down stairs) common between the Korean surveys (all four in the Census, only b140 in the KNHANES) and MDS that are not encompassed in the Generic-30 Set. The Generic-30 Set also does not contain three common categories found across the Korean surveys (d160 Focusing attention, d360 Using communication devices and techniques and d630 Preparing meals). As 70% of these categories address cognitive and communication aspects, aspects that are limited in the Generic-30, investigating the need for extending the Generic-30 Set to include more cognitive and communication categories may be warranted. Secondly, although we linked each question in the survey to the ICF Generic Set to confirm the information collected for each functioning category, we did not conduct statistical comparisons of the data collected in each survey. In particular, it is essential to compare the results of d450 walking, d510 washing oneself, and d540 dressing that are commonly assessed in the general population by the Census, KNHANES, and MDS. This comparison is important to ensure the reliability of the functioning information collected in Korean surveys. Furthermore, future studies should involve comparing survey results across different populations (e.g., the general population and registered persons with disabilities) and assessing statistical significance.

## Conclusion

Our study used the ICF as a reference framework to evaluate how functioning information is currently collected in Korea’s health and disability surveys, especially in terms of comparability across data sources and comprehensiveness of coverage of the ICF Generic-30. We confirm that Korean functioning information is collected periodically through national agencies, such as the Ministry of Health and Welfare and Statistics Korea, and different data sources are interoperable with other Korean and international data sources. An added value of our study is its potential to inform the generation of the functioning indicator as well as a potential Korean common health metric based on functioning which allows for the comparison of populations addressed in different surveys, for instance between the general Korean population and persons with disabilities or persons with SCI living in Korea. Additionally, this functioning metric can serve as valuable data for estimating and monitoring rehabilitation or long-term care needs of the ageing Korean population.
